# Norwegian Biological Parents and Stepparents’ Attitudes Towards Family Obligations in Middle and Old Age

**DOI:** 10.1177/0192513X241236563

**Published:** 2024-02-27

**Authors:** Julia Sauter

**Affiliations:** Norwegian Social Research, Oslo Metropolitan University, Oslo, Norway

**Keywords:** stepfamilies, intergenerational, remarriage, quantitative, parent/child relations

## Abstract

This study investigated the associations between the current family structure and the agreement with family obligations in middle and old age. It also tested whether gender differences exist in these associations. Based on research that has observed steprelationships tend to be less close than biological relationships are, it is argued that older individuals who are stepparents have lower agreement with family obligations than their counterparts in biological families have. The analytical sample was drawn from the Norwegian Life Course Ageing and Generation survey and consisted of 5564 individuals aged 50 and older. Findings suggest that individuals who do not have biological children but who are stepparents to their partners’ children agree more with filial obligations and less with parental obligations than biological parents do. The findings extend knowledge on diverse family structures in old age.

Over recent decades, and as the result of rising divorce and remarriage rates, family structures have diversified and become increasingly complex. Because of this complexification of family relationships, an increasing number of older adults are no longer part of biological families but are embedded in stepfamily structures (Bengtson, 2001; Dykstra & Hagestad, 2007; Thomson, 2014). Several international studies have produced evidence indicating that divorce, widowhood, and remarriage have detrimental effects on intergenerational relationships in later life (Aquilino, 1994; Ganong & Coleman, 2006; Kalmijn, 2008; Steinbach & Hank, 2016). Negative, or at least ambivalent, relationships can reduce the support older adults can potentially use in later years (Sherman et al., 2013; Willson et al., 2003). Attitudes towards family obligations are often seen as a precursor to supportive behaviors towards family members (Fazio, 1986). *Family obligations* refers to rules defined in cultural settings that determine which family members should support each other (Rossi & Rossi, 2018). In the present study, this translates to attitudes towards statements such as “Parents should get help from adult children” but also “If adult children need help, parents should adapt their lives to help them.” While the first example illustrates a filial obligation, meaning an attitude from adult children towards their older parents, the second example illustrates a parental obligation, meaning an attitude from older parents towards their adult children. By differentiating between complex and simple stepfamilies and biological families, this study investigates the degree to which older adults in nontraditional family structures agree with filial and parental obligations. In addition, I investigate whether gender differences exist in these associations.

## Theoretical Background

### Family Obligations

Family solidarity and family obligations have been the object of research for family sociologists since several decades. Emphasizing the significance of family obligations is crucial as they serve as indicators of a family’s true functionality. Initially, the impact of feelings of obligation on the provision of support is noteworthy (Ikkink et al., 1999; Stein et al., 1998). The stronger the sense of obligation a family member harbors to offer specific support, the greater the likelihood of them actually providing assistance when needed. Additionally, feelings of obligation serve as a vital benchmark for family members to assess tangible support exchanges within the family unit. Individuals gauge the support they receive from family members based on what they perceive these relatives are obligated to provide (Lee et al., 1994). To explain agreement with family obligations, this study mobilizes elements from the theory of intergenerational solidarity (Bengtson & Roberts, 1991), namely, normative solidarity. *Intergenerational solidarity* is defined as social cohesion between generations (Bengtson, 1975). Bengtson and Roberts (1991) identified six dimensions of intergenerational solidarity: associational, affectual, consensual, functional, structural, and normative solidarity. In this study, I focus on normative solidarity, which is defined as the “strength of commitment to performance of familial roles and to meeting familial obligations” (Bengtson & Roberts, 1991, p. 857). Helping behaviors in family relationships are based on widely held expectations about what should be done between family members (Finch & Mason, 1993; Lee & Shehan, 1989). Normative attitudes toward family obligations function as parameters within which individuals define and negotiate their responsibilities. They also provide a framework that people use to justify and explain their actions towards others, and they act as guidelines for individual behaviors (Finch, 1987). However, the strength of obligation highly depends on the type of family relationship. For instance, within the family structure, norms exhibit the highest strength when directed towards both parents and children, establishing them as the primary focal points. Siblings secure the third position in this hierarchy, while the fourth rank is collectively assigned to grandchildren, grandparents, children-in-law, and parents-in-law. In contrast, feelings of obligation are notably diminished when directed towards stepchildren, stepparents, nieces and nephews, and further attenuate in relationships with aunts, uncles, and cousins, following a descending order of priority (Rossi & Rossi, 2018). Nevertheless, normative solidarity among families and the family obligations that are derived from it are still important indicators for overall social cohesion, although they vary between family cultures and welfare regimes (Bengtson, 2001; Blome et al., 2009; Silverstein & Giarrusso, 2010).

### Family Obligations and Boundary Ambiguity in Stepfamilies

Stepfamilies are characterized by a combination of biological and no biological ties. The sole reason a relationship between stepparent and stepchild exists is the relationship both have with a third person, namely, the child’s biological parent who is simultaneously the romantic partner of the stepparent (Ganong et al., 1999). The roles and expectations within stepfamilies are often ambiguous, creating more opportunities for conflict and disagreement (Hobart, 1991). Ambiguous family boundaries have been found to have an association with poorer family functioning (Amorim, 2023; Brown & Manning, 2009). Family boundary ambiguity refers to the lack of clarity or consensus among family members regarding their roles, relationships, and expectations within the stepfamily structure (Boss, 1980; Stewart, 2005). In stepfamilies, the blending of individuals with distinct histories, attachments, and affiliations often leads to unclear boundaries, making it challenging for family members to navigate their roles and establish a cohesive family identity (Stewart, 2005). This ambiguity can manifest in various ways, such as uncertainty about parenting responsibilities, ambiguous kinship ties, and the presence of unresolved loyalties to previous families.

Moreover, relationships in stepfamilies tend to be less close and less frequent in their contact than relationships in biological families (Steinbach & Hank, 2016; Bildtgård & Öberg, 2022). They have also been found to be characterized by a lack of support transfers (Wiemers et al., 2018). Given this lack of closeness and the lack of a biological tie, the ambiguity of roles in stepfamilies has been linked to lower levels of agreement with filial and parental obligations in stepfamilies in the past, although most studies have focused on the viewpoint of adult stepchildren rather than older stepparents (Clawson & Ganong, 2002; Coleman et al., 2005; Ganong & Coleman, 2014; van Houdt et al., 2018). To have a more nuanced overview of different stepfamily structures, the present study differentiates between simple and complex stepfamilies. Simple stepfamilies consist of older adults who do not have children of their own, but are stepparents to their partner’s child(ren). Complex stepfamilies are constituted by older adults who have at least one biological child from a previous relationship but who are also stepparents to their partner’s child(ren). This distinction allows to account for the complexity in stepfamily relationships where different roles are often fulfilled by the same individual (Henderson & Taylor, 1999; Steinbach & Hank, 2016).

### Gender Differences in Steprelationships and Role Expectations

Roles and role expectations in different family structures are not the same for each gender (Seelbach, 1977; Silverstein et al., 1995). Research has shown that stepfathers more strongly perceive their stepchildren as their own than stepmothers do (Marsiglio, 2004; van Houdt, 2021a), and that adult children and stepchildren are more often detached from stepmothers than stepfathers (van Houdt, 2021b). Research has explained this discrepancy between genders as a result of different role expectations for stepfathers and stepmothers. Although stepfathers define their role primarily in terms of providing and authority, stepmothers see their role expectations as homemakers and kin keepers, which translates into an active, involved role in the stepchildren’s lives. Therefore, the requirements of involvement in stepchildren’s lives are more easily met by stepfathers than by stepmothers (Erera-Weatherley, 1996; Svare et al., 2004). The present study investigates further whether differences exist between mothers and stepmothers and fathers and stepfathers in nontraditional family structures in their association with agreement or disagreement with family obligations.

### Family Obligations in the Norwegian Context

Even though Norway is known for its generous and extensive welfare state policies (Bean & Papadakis, 1998; Linos & West, 2003; Svallfors, 1997), family remains an important aspect of society and an important pillar for the provision of assistance to older people (Daatland, 1997). Studies on the prevalence of filial obligations in biological families in Norway have shown rather strong levels of agreement with those norms across different age groups (Daatland et al., 2012; Daatland & Herlofson, 2003). Moreover, Daatland and colleagues (2012) found that Norwegian biological parents tend to have higher scores on parental obligations and lower scores on filial obligations compared to adult children. However, not much is known about the associations between stepfamily structures and the agreement to family obligations by stepparents in the context of Norway (Syltevik, 2017).

Despite the prevalence of the biological family still being high, the demographic trends towards higher numbers of complex family structures are clearly present in Norway, with high rates of divorce and remarriage across all age groups (Zahl-Olsen et al., 2019). In 2018, 28% of married people were in at least their second marriage (Zahl-Olsen, 2023). Experiencing divorce and remarriage is associated with potentially becoming a stepparent. Nevertheless, Norwegian stepfamilies, especially in old age, have not yet received much attention from researchers. To the best of my knowledge, no prior international quantitative study has investigated the prevalence of family obligations in different family contexts in Norway. Therefore, this study explores the associations between simple and complex stepfamilies and agreement with family obligations using data from older Norwegian adults.

### Summary

The associations between stepfamily structures and family obligations have been investigated. However, most studies have been from the viewpoint of adult children or stepchildren rather than older parents or stepparents (Coleman et al., 2005; Ganong & Coleman, 1998; van Houdt et al., 2018), have used qualitative data (Clawson & Ganong, 2002), or have focused on support (Kalbarczyk, 2021) or relationship quality (Steinbach & Hank, 2016) rather than family obligations. Therefore, the present study extends the literature on the associations between family structures and family obligations from the viewpoint of parents and stepparents using a large quantitative sample of older adults.

Given the increased role ambiguity in stepfamilies compared to biological families, I expect that stepparents in simple and complex stepfamilies will have lesser levels of agreement with filial obligations than parents in biological families (Hypothesis 1a). Moreover, given that parents in complex stepfamilies are biological and stepparents simultaneously, I expect the association to be less strong than in simple stepfamilies, in which individuals only hold the role of stepparents (Hypothesis 1b). With regards to parental obligations, I also expect that stepparents in simple and complex stepfamilies have lesser levels of agreement with parental obligations than parents in biological families (Hypothesis 2a). Here again, given that parents in complex stepfamilies are biological and stepparents simultaneously, I expect the association to be less strong in complex stepfamilies than in simple stepfamilies, in which individuals only hold the role of stepparents (Hypothesis 2b). Furthermore, I hypothesize that stepfathers agree more with filial and parental obligations than stepmothers (Hypothesis 3), because stepmothers are often less close with their stepchildren and are held to higher standards of providing parental support than are stepfathers.

## Methods

### Data

Data came from the third wave of the Norwegian Life Course, Ageing, and Generation Study (NorLAG) conducted in 2017. NorLAG was initiated in 2002 to gain new and updated knowledge on aging and age-related changes in Norwegian society. The second wave was conducted in 2007 and consisted of 9238 respondents, of which 6099 were interviewed again in 2017 (Veenstra et al., 2021). The sample for the present study consists of 5564 participants who were aged 50 or older at the time of interview and who provided information on their family structures and answered questions about their perception of family obligations.

### Dependent Variables

I included the perception of six family obligations as dependent variables in the analysis. Four of the dependent variables indicate children’s normative attitudes towards their parents (filial obligations) and two indicate parents’ attitudes towards their children (parental obligations). Both types of obligation are deeply intertwined (Dykstra & Fokkema, 2011; Fokkema et al., 2008). In other words, when normative solidarity towards aging parents is strong, older parents also feel a stronger normative obligation towards their adult children. The four perceptions of filial obligations include the following statements: “Adult children should live close to their parents”; “Grown children should make sacrifices for their parents”; “Parents should get help from adult children”; and “Parents should get something back from their children.” The two perceptions of parental obligations include the following statements: “Parents should leave something for their descendants” and “If adult children need help, parents should adapt their lives to help them.” Participants were asked to indicate their agreement to those statements on a 5-point Likert scale ranging from 1 (*fully agree*) to 5 (*fully disagree*). Answers were then recoded into three categories, namely, 1 (*agree*), 2 (*indifferent*), and 3 (*disagree*).

### Independent Variable

The independent variable for the present study is participants’ present family structure. I divided this variable into three categories: simple stepfamily, complex stepfamily, and biological family. To categorize participants into family structures, I crossed information on having biological children and having stepchildren. I included biological and stepchildren regardless of whether they lived with the respondent. Participants in simple stepfamilies were those who did not have their own children but whose partners had children from previous relationships. The complex stepfamily category included respondents who had both their own children and stepchildren, whereas biological families consisted of those participants who only had their own children. Participants who did not have their own children and who either lived alone or with partners who also did not have children from previous relationships were not included in the analysis.

### Control Variables

I included the following six variables as controls in the analysis. First, I controlled for gender (1 = men, 2 = women). Second, I controlled for respondents’ age group (10-year intervals) at the time of interview. The choice of including age group rather than age in years was made because including age in years would have led to a considerable percentage (on average 50%) of cells with zero frequencies (i.e., dependent variable levels by observed combinations of predictor variable values). By including age group, I could reduce this to around 20%. I also controlled for level of education, using three categories, namely, low (1 = compulsory education or no education), average (2 = secondary education), and high (3 = university or equivalent). Prior studies have shown that higher educational levels were associated with greater agreement to family obligations (Valkde & Schans, 2008; Daatland et al., 2011). I also controlled for whether participants’ parents were alive (0 = no, 1 = yes) and if they coresided with their children or stepchildren at the time of the data collection (0 = coresidence, 1 = no coresidence). Controlling for the fact if parents are still alive allows to take into account participants’ generational placement (i.e., being a parent while being also a child vs. only being a parent). The impact of generational placement on support provided to older adults and adult children has been investigated in the past (Silverstein et al., 2020), however, evidence on the potential impact on agreement to family obligations is still limited. The choice of controlling for coresidence was made because coresidence was shown to be highly correlated to higher levels of agreement to parental and filial obligations in the past (van Houdt et al., 2018).

### Statistical Analysis

I first present descriptive statistics for the sample in terms of sociodemographic profile in each family structure. I ran multinomial logistic regression models to test for the association between family structure and family obligations. I chose to perform separate multinomial logistic regression models for each family obligation norm rather than combining them into an aggregate measure because I argue that these different statements reflect different dimensions of family attitudes. This is also in line with research that has analyzed different family obligation norms separately (Burr & Mutchler, 1999; Valkde & Schans, 2008). Moreover, I tested two models for each outcome variable. The first only tested for the association between family structure and family obligation, whereas the second includes an interaction effect between family structure and gender. Including this interaction effect allows testing for potential gender differences between family structures in their association with family obligations. It should be noted that on average 20% of the cells in all models had zero frequencies (i.e., dependent variable levels by observed combinations of predictor variable values).

## Results

### Descriptive Statistics

Respondents’ sociodemographic profiles for each family structure are presented in Table 1. Most respondents (85.5%) were in biological families, whereas only 2% were in simple stepfamilies and 12.6% were in complex stepfamilies. Of simple stepfamilies, 41.1% consisted of stepmothers. In complex stepfamilies, 46.9% and in biological families 51.6% were women. The differences in the share of women in the three family structures were significant. In the simple stepfamilies, no stepparents coresided with their stepchildren at the time of interview, whereas 17.1% of individuals in complex stepfamilies and 17.7% of individuals in biological families coresided with at least one child or stepchild. Most participants in all three family structures were either in the middle- or high-education category, and the differences in level of education between family structures were not significant.

**Table 1.** Descriptive Statistics on Sociodemographic Variables by Family Structure.

Variable	Categories	Simple stepfamily	Complex stepfamily	Biological family	χ^2^
		%	%	%	
Age groups					χ^2^ = 87.93***
	50–59	44.9	39.9	31.7	
	60–69	37.4	40.7	33.2	
	70–79	15.9	15.8	25.0	
	≥80	1.9	3.6	10.2	
Education					χ^2^ = 7.24
	Low	10.3	13.6	16.0	
	Mid	44.9	47.7	47.7	
	High	44.9	38.7	36.4	
Gender	Women	41.1	46.9	51.6	χ^2^ = 9.38**
Coresidence with children or stepchildren	Yes	0.0	17.1	17.7	χ^2^ = 22.86***
Parents alive	Yes	14.0	16.2	13.2	χ^2^ = 4.83
*N*		107 (1.9%)	700 (12.6%)	4757 (85.5%)	5564 (100%)

*Note.* *p < .05, **p < .01, ***p < .001.

### Multinomial Logistic Regression Analysis

Table 2 shows the results of the multinomial logistic regression models for the four filial obligations under investigation, comparing participants who agreed with those who were indifferent. No statistically significant associations between family structures and family obligation were found for the first two statements, namely “Adult children should live close to their parents” and “Grown children should make sacrifices for their parents.” For the statement “Parents should get help from adult children,” the model including the interaction indicated that individuals in simple stepfamilies were more likely to agree rather than to be indifferent compared to individuals in biological families. Moreover, the interaction effect showed a negative association. Therefore, although women in simple stepfamilies agreed more, men in simple stepfamilies were more indifferent. In other words, a gender difference exists in the association between simple stepfamilies and agreement with this statement.

**Table 2.** Multinomial Logistic Regression Analysis on Filial Obligations (Agree Compared to Indifferent).

Predictors	Adult children should live close to their parents		Grown children should make sacrifices for their parents		Parents should get help from adult children		Parents should get something back from their children	
	Model 1	Model 2	Model 1	Model 2	Model 1	Model 2	Model 1	Model 2
Family structure (ref.: biological family)								
Simple stepfamily	0.70 [0.36, 1.36]	1.34 [0.52, 3.41]	1.43 [0.88, 2.33]	1.42 [0.69, 2.94]	1.30 [0.79, 2.13]	2.75* [1.19, 6.33]	1.28 [0.70, 2.35]	0.85 [0.28, 2.59]
Complex stepfamily	1.12 [0.87, 1.49]	1.25 [0.84, 1.87]	1.00 [0.80, 1.26]	0.82 [0.59, 1.15]	1.07 [0.85, 1.34]	0.96 [0.69, 1.33]	1.45* [1.08, 1.95]	0.69 [0.39, 1.23]
Control variables								
Male (ref.: female)	1.40*** [1.17, 1.67]	1.46*** [1.20, 1.78]	1.40*** [1.21, 1.63]	1.34*** [1.14, 1.57]	1.25** [1.08, 1.46]	1.26** [1.06, 1.48]	1.69*** [1.37, 2.08]	1.44** [1.15, 1.81]
Age group (ref.: >80)								
50–59	0.43*** [0.30, 0.61]	0.43*** [0.30, 0.61]	0.70* [0.51, 0.96]	0.70* [0.51, 0.96]	0.86 [0.62, 1.19]	0.86 [0.62, 1.19]	0.78 [0.52, 1.19]	0.78 [0.51, 1.19]
60–69	0.58*** [0.42, 0.78]	0.58*** [0.43, 0.79]	0.63*** [0.47, 0.83]	0.62*** [0.47, 0.83]	0.76 [0.57, 1.02]	0.77 [0.57, 1.03]	0.87 [0.59, 1.27]	0.85 [0.58, 1.25]
70–79	0.57*** [0.42, 0.79]	0.58*** [0.42, 0.79]	0.51*** [0.38, 0.69]	0.51*** [0.38, 0.69]	0.69* [0.51, 0.94]	0.69* [0.51, 0.94]	0.77 [0.52, 1.16]	0.76 [0.51, 1.14]
Parents deceased (ref.: alive)	0.83 [0.62, 1.12]	0.83 [0.62, 1.12]	0.80 [0.63, 1.01]	0.81 [0.64, 1.02]	0.82 [0.65, 1.05]	0.83 [0.65, 1.06]	0.93 [0.68, 1.27]	0.94 [0.68, 1.29]
Education (ref.: high)								
Mid	1.08 [0.89, 1.32]	1.09 [0.89, 1.32]	0.76*** [0.65, 0.89]	0.76*** [0.65, 0.89]	0.89 [0.75, 1.04]	0.89 [0.75, 1.05]	1.18 [0.95, 1.48]	1.18 [0.95, 1.48]
Low	1.60*** [1.22, 2.10]	1.61*** [1.23, 2.11]	0.88 [0.69, 1.12]	0.88 [0.69, 1.12]	1.17 [0.91, 1.49]	1.18 [0.93, 1.51]	1.32 [0.97, 1.80]	1.32 [0.97, 1.80]
Coresidence with children or stepchildren (ref.: empty-nest)	1.12 [0.85, 1.48]	1.12 [0.85, 1.48]	1.04 [0.83, 1.30]	1.05 [0.84, 1.31]	1.16 [0.92, 1.46]	1.16 [0.92, 1.46]	1.16 [0.86, 1.56]	1.17 [0.87, 1.58]
Interaction family structure × Gender								
Simple stepfamily × Male		0.32 [0.08, 1.22]		1.03 [0.39, 2.71]		0.29* [0.10, 0.82]		1.91 [0.51, 7.23]
Complex stepfamily × Male		0.82 [0.48, 1.40]		1.44 [0.92, 2.25]		1.23 [0.78, 1.93]		2.89** [1.48, 5.68]
*N*	3918	3918	3907	3907	3918	3918	3893	3893
Nagelkerke’s Pseudo *R*^2^	0.04	0.04	0.04	0.04	0.03	0.03	0.05	0.06

*Note.* Values given are odds ratios with 95% confidence intervals in brackets.

*p < .05, **p < .01, ***p < .001.

Table 3 shows the results of the multinomial logistic regressions for the two parental obligations included in the present study, comparing those who agreed with those who were indifferent. For the first statement of parental obligation, namely, “Parents should leave something for their descendants,” individuals who were in one of the two nontraditional family structures (simple and complex stepfamilies) were less likely to agree than those in biological families. However, when introducing the interaction term, these effects were no longer significant. For the second statement of parental obligation, namely, on parents helping their adult children when needed, individuals who were in one of the nontraditional family structures (simple and complex stepfamilies) were less likely to agree than those in biological family structures. Here again, when introducing the interaction term, these effects were no longer significant.

**Table 3.** Multinomial Logistic Regression Analysis on Parental Obligations (Agree Compared to Indifferent).

Predictors	Parents should leave something for their descendants		If adult children need help, parents should adapt their lives to help them	
	Model 1	Model 2	Model 1	Model 2
Family structure (ref.: biological family)				
Simple stepfamily	0.34** [0.17, 0.69]	0.26 [0.06, 1.18]	0.44* [0.22, 0.85]	0.70 [0.22, 2.26]
Complex stepfamily	0.78* [0.61, 1.00]	0.93 [0.63, 1.37]	0.74* [0.57, 0.95]	0.82 [0.55, 1.21]
Control variables				
Male (ref.: female)	1.56*** [1.33, 1.83]	1.62*** [1.37, 1.93]	1.76*** [1.51, 2.07]	1.82*** [1.54, 2.16]
Age group (ref.: >80)				
50–59	0.54*** [0.39, 0.75]	0.54*** [0.39, 0.75]	0.72* [0.52, 1.00]	0.72* [0.52, 1.00]
60–69	0.69* [0.52, 0.93]	0.69* [0.52, 0.93]	0.78 [0.58, 1.04]	0.78 [0.58, 1.05]
70–79	0.69* [0.51, 0.93]	0.69* [0.52, 0.93]	0.94 [0.69, 1.27]	0.94 [0.70, 1.28]
Parents deceased (ref.: alive)	1.24 [0.94, 1.63]	1.24 [0.94, 1.63]	1.21 [0.92, 1.60]	1.22 [0.92, 1.60]
Education (ref.: high)				
mid	0.79** [0.66, 0.93]	0.79** [0.66, 0.93]	0.70*** [0.59, 0.83]	0.70*** [0.59, 0.83]
low	0.76* [0.58, 0.98]	0.76* [0.58, 0.98]	0.64*** [0.49, 0.82]	0.64*** [0.49, 0.83]
Coresidence with children or stepchildren (ref.: empty-nest)	1.08 [0.84, 1.39]	1.08 [0.84, 1.38]	0.85 [0.66, 1.09]	0.85 [0.66, 1.09]
Interaction family structure × Gender				
Simple stepfamily × Male		1.34 [0.24, 7.35]		0.49 [0.12, 2.01]
Complex stepfamily × Male		0.74 [0.44, 1.22]		0.84 [0.50, 1.40]
*N*	3897	3897	3870	3870
Nagelkerke’s Pseudo *R*^2^	0.07	0.07	0.07	0.07

*Note.* Values given are odds ratios with 95% confidence intervals in brackets.

*p < .05, **p < .01, ***p < .001.

Table 4 shows the results of the multinomial logistic regression models for the four filial obligations under investigation, comparing participants who disagreed with those who were indifferent. For all four statements of filial obligations, individuals in simple stepfamilies were more likely to be indifferent than those in biological families. In the models that included the interaction effect between family structure and gender, these effects remained significant for the statements “Grown children should make sacrifices for their children” and “Parents should get something back from their children.” However, none of the interaction effects were significant, showing no gender differences between family structures.

**Table 4.** Multinomial Logistic Regression Analysis on Filial Obligations (Disagree Compared to Indifferent).

Predictors	Adult children should live close to their parents		Grown children should make sacrifices for their parents		Parents should get help from adult children		Parents should get something back from their children	
	Model 1	Model 2	Model 1	Model 2	Model 1	Model 2	Model 1	Model 2
Family structure (ref.: Biological family)								
Simple stepfamily	0.54* [0.32, 0.91]	0.63 [0.29, 1.36]	0.43* [0.21, 0.90]	0.23* [0.07, 0.82]	0.54 [0.29, 1.02]	0.83 [0.30, 2.32]	0.56* [0.34, 0.92]	0.44* [0.22, 0.91]
Complex stepfamily	1.14 [0.93, 1.40]	1.25 [0.94, 1.66]	1.07 [0.85, 1.36]	1.01 [0.74, 1.38]	1.07 [0.84, 1.34]	1.07 [0.78, 1.46]	1.21 [0.97, 1.50]	1.16 [0.86, 1.57]
Control variables								
Male (ref.: female)	0.86* [0.75, 0.99]	0.89 [0.76, 1.03]	0.75*** [0.63, 0.88]	0.73*** [0.61, 0.87]	0.86* [0.73, 1.00]	0.87 [0.73, 1.03]	0.64*** [0.55, 0.74]	0.63*** [0.54, 0.74]
Age group (ref.: >80)								
50–59	2.21*** [1.55, 3.16]	2.21*** [1.55, 3.16]	1.83** [1.24, 2.70]	1.83** [1.24, 2.70]	1.53* [1.06, 2.20]	1.53* [1.06, 2.20]	1.25 [0.92, 1.71]	1.25 [0.92, 1.71]
60–69	2.13*** [1.52, 2.98]	2.14*** [1.53, 3.00]	1.47* [1.03, 2.12]	1.47* [1.02, 2.11]	1.35 [0.96, 1.89]	1.35 [0.97, 1.90]	1.28 [0.96, 1.70]	1.28 [0.96, 1.70]
70–79	2.11*** [1.49, 2.98]	2.12*** [1.50, 2.99]	1.38 [0.95, 2.00]	1.38 [0.95, 2.00]	1.50* [1.06, 2.12]	1.50* [1.06, 2.12]	1.54** [1.14, 2.06]	1.53** [1.14, 2.06]
Parents deceased (ref.: alive)	1.04 [0.83, 1.30]	1.04 [0.83, 1.30]	1.35* [1.03, 1.77]	0.81 [0.64, 1.02]	1.16 [0.89, 1.51]	1.16 [0.89, 1.51]	1.12 [0.89, 1.40]	1.11 [0.89, 1.40]
Education (ref.: high)								
mid	0.87 [0.75, 1.01]	0.87 [0.75, 1.01]	0.91 [0.77, 1.08]	0.76*** [0.65, 0.89]	0.83* [0.70, 0.98]	0.83* [0.70, 0.98]	0.89 [0.77, 1.04]	0.89 [0.77, 1.04]
low	0.84 [0.67, 1.07]	0.85 [0.67, 1.07]	0.91 [0.70, 1.18]	0.88 [0.69, 1.12]	0.87 [0.67, 1.13]	0.87 [0.68, 1.13]	0.65*** [0.51, 0.81]	0.64*** [0.51, 0.81]
Coresidence with children or stepchildren (ref.: empty-nest)	0.80* [0.64, 0.99]	0.80* [0.64, 0.99]	0.80 [0.63, 1.03]	1.05 [0.84, 1.31]	0.89 [0.69, 1.14]	0.89 [0.70, 1.14]	0.86 [0.69, 1.06]	0.85 [0.69, 1.06]
Interaction family structure × Gender								
Simple stepfamily × Male		0.77 [0.27, 2.19]		1.03 [0.39, 2.71]		0.52 [0.14, 1.92]		1.54 [0.57, 4.13]
Complex stepfamily × Male		0.84 [0.55, 1.26]		1.44 [0.92, 2.25]		0.99 [0.62, 1.58]		1.05 [0.69, 1.62]
*N*	3918	3918	3907	3907	3918	3918	3893	3893
Nagelkerke’s Pseudo *R*^2^	0.04	0.04	0.04	0.04	0.03	0.03	0.05	0.06

*Note.* Values given are odds ratios with 95% confidence intervals in brackets.

*p < .05, **p < .01, ***p < .001.

Table 5 shows the results of the multinomial logistic regressions for the two parental obligations included in the present study, comparing those who disagreed with those who were indifferent. For the first statement of parental obligations, namely, “Parents should leave something for their descendants,” individuals in complex stepfamilies were more likely to disagree than those in biological families. These effects remained significant in the interaction model, whereas the interaction terms were not significant. For the second statement, “If adult children need help, parents should adapt their lives to help them,” individuals in complex stepfamilies were more likely to disagree than individuals in biological families. In the interaction model, the association between simple stepfamily structures and disagreement with the above mentioned obligation was significant as well. However, none of the interaction effects were significant for either of the statements.

**Table 5.** Multinomial Logistic Regression Analysis on Parental Obligations (Disagree Compared to Indifferent).

Predictors	Parents should leave something for their descendants		If adult children need help, parents should adapt their lives to help them	
	Model 1	Model 2	Model 1	Model 2
Family structure (ref.: Biological family)				
Simple stepfamily	1.05 [0.65, 1.69]	1.40 [0.69, 2.87]	1.25 [0.77, 2.05]	2.16* [0.99, 4.70]
Complex stepfamily	1.25* [1.01, 1.55]	1.49** [1.10, 2.01]	1.44*** [1.16, 1.78]	1.47* [1.09, 1.97]
Control variables				
Male (ref.: female)	0.65*** [0.56, 0.76]	0.70*** [0.59, 0.82]	0.80** [0.68, 0.93]	0.82* [0.69, 0.97]
Age group (ref.: >80)				
50–59	2.24*** [1.55, 3.25]	2.25*** [1.55, 3.26]	2.18*** [1.51, 3.14]	2.18*** [1.51, 3.14]
60–69	1.85*** [1.30, 2.63]	1.86*** [1.31, 2.65]	1.51* [1.07, 2.13]	1.52* [1.08, 2.14]
70–79	1.69** [1.17, 2.42]	1.70** [1.18, 2.44]	1.51* [1.06, 2.16]	1.52* [1.06, 2.17]
Parents deceased (ref.: alive)	1.01 [0.80, 1.27]	1.00 [0.79, 1.27]	0.89 [0.71, 1.13]	0.89 [0.71, 1.13]
Education (ref.: High)				
mid	1.27** [1.08, 1.49]	1.27** [1.08, 1.50]	1.21* [1.02, 1.43]	1.21* [1.03, 1.43]
low	1.21 [0.95, 1.53]	1.21 [0.95, 1.54]	1.01 [0.79, 1.29]	1.02 [0.79, 1.30]
Coresidence with children or stepchildren (ref.: empty-nest)	0.98 [0.78, 1.23]	1.00 [0.79, 1.27]	0.97 [0.78, 1.22]	0.98 [0.78, 1.22]
Interaction family structure × Gender				
Simple stepfamily × Male		0.57 [0.22, 1.50]		0.38 [0.14, 1.06]
Complex stepfamily × Male		0.70 [0.45, 1.08]		0.96 [0.63, 1.48]
*N*	3897	3897	3870	3870
Nagelkerke’s Pseudo *R*^2^	0.07	0.07	0.07	0.07

*Note.* Values given are odds ratios with 95% confidence intervals in brackets.

*p < .05, **p < .01, ***p < .001.

## Discussion

The aim of this study was to investigate the extent to which older adults in complex and simple stepfamilies agreed with attitudes towards family solidarity compared to biological families. I used a large representative sample of older Norwegian parents and stepparents who had only stepchildren, only their own children, or both. Based on research on family obligations in stepfamilies (Coleman et al., 2005; Ganong & Coleman, 1998, 2014; van Houdt et al., 2018), but also extending the literature by considering the viewpoint of aging parents and stepparents rather than adult children and stepchildren, this study hypothesized that individuals in simple and complex stepfamilies had lower levels of agreement with attitudes towards filial responsibility than individuals in biological families (Hypothesis 1a) and that said effects would be even greater in simple stepfamilies than in complex stepfamilies (Hypothesis 1b). The present study did not confirm these hypotheses. On the contrary, the results show that stepparents in stepfamilies, meaning individuals who do not have biological children but have stepchildren through their current partner, agreed more with statements of filial obligations than individuals in biological families did.

Several factors can explain these results. First, stepparents without biological children may tend to have higher expectations towards their stepchildren than a biological parent has because they only have limited or no experience in parenting (Craig et al., 2012). Although most individuals who are either only biological or biological and stepparents have experienced decades of parenting, conflicts, exchanges, and coresidence with their now adult children, childless stepparents might only be a recent part of their current stepfamily. This in turn might lead to childless stepparents who tend to have a different perspective on how their partner should be treated by and what they should receive from their children.

From the viewpoint of the childless stepparent, their partner might not receive enough support from their adult children and therefore the stepparent tends to agree more with those attitudes of filial obligations. This finding can also be partly explained by the wording of the different attitudes. Each statement on filial obligations explicitly referred to “parents” rather than “parents or stepparents.” It is therefore likely that childless stepparents did not project themselves in the statements but rather their partner who is the biological parent. This explanation is also in line with the role and family boundary ambiguity found in most stepfamilies, where stepparents do not consider themselves as parents to their stepchildren per se (Castrén & Widmer, 2015; Craig et al., 2012; Stewart, 2005). Indeed, the theory on family boundary ambiguity indicates that the lack of clarity or consensus among family members regarding their roles, relationships, and expectations within the stepfamily structure (Boss, 1980; Stewart, 2005).

This study further hypothesized that individuals in simple and complex stepfamilies had lower levels of agreement with parental obligation norms than individuals in biological families had (Hypothesis 2a) and that these effects would be greater for individuals in simple stepfamilies (Hypothesis 2b). The first hypothesis was confirmed by the results, but not the second. For both statements included in the analysis, namely, “Parents should leave something for their descendants” and “Parents should adapt their lives to help adult children,” individuals in simple and complex stepfamily structures agreed less than individuals in biological families. However, contrary to my expectations, these associations were greater for individuals in complex stepfamilies than for those in simple stepfamilies. These findings are in line with previous findings showing that steprelationships are associated with fewer support transfers from stepparents to their stepchildren (Wiemers et al., 2018), less relationship closeness (Becker et al., 2013; Steinbach & Hank, 2016), and less contact (van der Pas & van Tilburg, 2010). It is therefore not surprising that parents in stepfamilies agreed less with parental obligations than did their counterparts in biological families. In the context of stepfamilies, the challenges associated with establishing clear family boundaries and navigating complex relationships contribute to decreased support transfers from stepparents to their stepchildren, diminished relationship closeness, and reduced contact, as observed in prior studies (Becker et al., 2013; Steinbach & Hank, 2016; van der Pas & van Tilburg, 2010; Wiemers et al., 2018). Consequently, the diminished agreement with parental obligations among parents in stepfamilies, compared to their counterparts in biological families, can be understood as a manifestation of the intricate dynamics inherent in stepfamily structures, emphasizing the importance of considering family systems theory in comprehensively elucidating these phenomena.

With regards to gender differences in the agreement with family obligations within different family structures, I hypothesized that stepfathers would agree more with filial and parental obligations than stepmothers would (Hypothesis 3). This hypothesis was only confirmed for one of the four filial obligations statements, namely, “Parents should get something back from their children.” For this statement, fathers and stepfathers in complex stepfamilies significantly agreed more than their female counterparts did. This association is in line with research that has shown higher levels of closeness between stepfathers and their stepchildren than stepmothers have (Marsiglio, 2004; van Houdt, 2021a). However, the results on gender differences in this study are rather limited, because no other significant differences between stepfathers and stepmothers were found.

Overall, the present study confirms that role ambiguity in stepfamilies translates into different levels of agreement with filial and parental obligations compared to biological families (Fine et al., 1998; Pasley & Ihinger-Tallman, 1989). Stepfamilies are indeed less institutionalized than biological families are, and they are therefore less clear in their family boundaries and therefore in the responsibilities and commitments that family members hold (Boss, 1980; Carroll et al., 2007; Golish, 2003; Stewart, 2005). As a result of this heightened ambiguity and in the absence of an institutional framework in which they could operate, stepfamilies navigate in a space in which parameters other than blood relationships and kinship define the existence of relationships. The results indirectly confirm this by showing that stepparents (especially those without their own children) have a different view on the ways family members should behave and what can be expected from stepfamily members than parents in biological families.

These findings also have policy implications. For instance, in light of population aging, a growing public concern is how societies should cope with the increasing demand for care for older adults (United Nations Department of Economic and Social Affairs, 2015). This demand also concerns individuals who live in nontraditional family forms, such as stepfamilies. The present study’s findings show the importance for policymakers to recognize and acknowledge the diversity of family structures and to understand the variations in agreement to family obligations across these different family structures, which are considered as precursors to later support behaviors. This is of heightened importance in the context of Norway, in which family is still considered as a main source of provision of informal support to older adults. Moreover, the gender differences observed in the associations between family structures and certain family obligations suggest that policies related to family support and intergenerational relationships may need to consider gender-specific dynamics. For instance, interventions or support programs may need to be tailored to address the specific needs of men and women in different family structures.

## Limitations and Future Research

This study has several limitations. First, this study does not consider participants’ family histories. The available data only allow us to determine the family structure in which individuals lived at the moment of the interview. The data do not have information on how long the steprelationships had existed, nor does it include information on prior living arrangements (e.g., did the respondents and their stepchildren live together in the past, and if so, for how long?) It is therefore not possible to give information on past events that might affect the ways respondents perceived filial and parental obligations. Nevertheless, I was able to establish whether stepchildren were present and therefore the family structure in which older adults lived at the moment of the survey. Second, the explained variance of the statistical models is rather small. Large parts of the variance (on average around 20% for each family obligation) were explained by prior level of agreement with family obligations assessed in the second wave of the NorLAG survey in 2007. However, the main goal of this study was to test whether an association exists between different family structures and family obligations without considering prior agreement to these obligations. I therefore argue that the results are still important and allow the advancement of knowledge in the field of stepfamily research.

Despite these shortcomings, the present study advances knowledge on the extent to which older adults who live in nontraditional family structures agree with norms of family obligations. Given that up until now only a few studies have focused on older stepparents’ perspectives on family obligations, future research should further investigate those issues with more detailed information on family histories. In conclusion, while this study enriches our understanding of family obligations in stepfamilies, it also underscores the need for ongoing critical examination of assumptions derived from previous literature. The unexpected findings challenge existing paradigms, emphasizing the need for a more nuanced understanding of the intricacies of stepfamily relationships. As the field evolves, researchers should continue to explore the multifaceted nature of family dynamics within stepfamilies, considering diverse family structures, individual experiences, and the potential impact of role ambiguity on perceptions of familial responsibilities.
